# A Small-Sized Population of Human Umbilical Cord Blood-Derived Mesenchymal Stem Cells Shows High Stemness Properties and Therapeutic Benefit

**DOI:** 10.1155/2020/5924983

**Published:** 2020-04-28

**Authors:** Miyeon Kim, Yun Kyung Bae, Soyoun Um, Ji Hye Kwon, Gee-Hye Kim, Soo Jin Choi, Wonil Oh, Hye Jin Jin

**Affiliations:** Biomedical Research Institute, MEDIPOST Co., Ltd., Seongnam 13494, Republic of Korea

## Abstract

Mesenchymal stem cells (MSCs) represent a promising means to promote tissue regeneration. However, the heterogeneity of MSCs impedes their use for regenerative medicine. Further investigation of this phenotype is required to develop cell therapies with improved clinical efficacy. Here, a small-sized population of human umbilical cord blood-derived MSCs (UCB-MSCs) was isolated using a filter and centrifuge system to analyze its stem cell characteristics. Consequently, this population showed higher cell growth and lower senescence. Additionally, it exhibited diverse stem cell properties including differentiation, stemness, and adhesion, as compared to those of the population before isolation. Using cell surface protein array or sorting analysis, both EGFR and CD49f were identified as markers associated with the small-sized population. Accordingly, suppression of these surface proteins abolished the superior characteristics of this population. Moreover, compared to that with large or nonisolated populations, the small-sized population showed greater therapeutic efficacy by promoting the engraftment potential of infused cells and reducing lung damage in an emphysema mouse model. Therefore, the isolation of this small-sized population of UCB-MSCs could be a simple and effective way to enhance the efficacy of cell therapy.

## 1. Introduction

Mesenchymal stem cells (MSCs) have been characterized according to stemness, ability to differentiate into various cell types, low immunogenicity and tumorigenicity, and the secretion of trophic factors. Based on these beneficial properties, MSCs have been extensively utilized for cell-based therapy [1]. However, they generally have been shown to comprise a heterogeneous mixture of different subpopulations. Importantly, the heterogeneity of MSCs is the result of various conditions including cell size, growth rate, morphology, differentiation potential, and senescence, leading to hurdles in the development of MSC-based therapy [2–4]. This heterogeneity limits a general understanding of the mechanism through which MSCs maintain their proliferative capacity and undergo differentiation toward specific lineage potentials, as well as approaches to achieve better outcomes with therapeutic applications. Heterogeneity is mainly affected by growth media, two-dimensional adherence to plastic dishes, and subculture methods within culture. However, this processing can be repeated to obtain an adequate number of MSCs for mass production.

In this context, many researchers have attempted to establish a standard set of criteria to attain more homogenous populations of MSCs. However, few studies have attempted to culture MSCs derived from a single cell or colony, and each original cell differs from each other [5–7]. Moreover, these obtained MSCs contain mixed populations exhibiting varying morphological features and gene expression patterns [8], which might imply that all cells are cultured in transitional culture environments. Recently, several groups have developed protocols to isolate more homogeneous cells from heterogeneous populations using specific antigens [9–11]; however, none of these processes have gained widespread acceptance, because there is no unique single marker. Other studies suggested cell seeding density or confluence as a major contributor to alterations in morphology and size [3, 12, 13]. However, to the best of our knowledge, these procedures have not been shown to affect MSC phenotypes. Despite such attempts, there is still no defined culture protocol available to overcome MSC heterogeneity.

Although cellular heterogeneity is caused by various factors, heterogeneous cells display a number of common characteristics that make them easily distinguishable based on cell size. The size of MSCs significantly increases during expansion. Importantly, senescent cells increase in cell size, sometimes enlarging more than twofold relative to the size of nonsenescent cells [14], which helps to explain some of the biological activities of senescent cells; SA *β*-gal activity and growth arrest [14]. Further, MSCs show heterogeneity in size as primary cells when cultured to senescence. Additionally, several studies have indicated an advantage using small cells from heterogeneous MSCs of the bone marrow (BM) or umbilical cord (UC) to enhance the function of stem cells due to the following reasons: (i) MSCs contain small spindle-shaped cells with rapid growth, whereas large or flat cells expand more slowly; (ii) the size of rapidly self-renewing cells or recycling stem cells is known to be in the range of ≤7~8 *μ*m or smaller in diameter; (iii) small cells (≤8~10 *μ*m diameter) have a greater potential for multilineage differentiation than populations enriched in large cells; and (iv) the small population (≤11 *μ*m diameter) exhibits faster growth and slower aging [15–17].

Small MSCs can be mainly separated through the following three methods: flow cytometric sorting, counter flow elution [17, 18], and microfluidic sorting [19]. However, no markers have been characterized for small cells. Moreover, to establish markers contributing to the formation of potentiated small MSCs, we screened the expression of surface proteins in small cells by fluorescence-activated cell sorting (FACS) analysis with 242 different cell surface antibodies. Among the examined surface proteins, we found the highest expression of epidermal growth factor receptor (EGFR) and integrin *α*6 (CD49f) in the small size population. In the present study, we aimed to analyze the stem cell characteristics of the small-sized population of human umbilical cord blood-MSCs (UCB-MSCs) using a filter and centrifuge system. The findings of our study provide evidence supporting the effect of this small population derived from UCB-MSCs and contribute new surface markers of small cells, which are contributors to MSC heterogeneity and of interest for therapeutic applications.

## 2. Methods

### 2.1. Cell Culture and Growth Kinetics

The UCB obtained from the umbilical vein after the neonatal delivery of an infant was processed within 24 h for the isolation and separation of mononuclear cells (MNCs) with Ficoll-Hypaque solution (density = 1.077 g/cm^3^; GE Healthcare, Uppsala, Sweden), followed by previous protocol [20, 21]. This protocol was approved by the Institutional Review Board of MEDIPOST Co., Ltd. (MP-2014-07-1-1). MNCs were washed with phosphate buffer saline (PBS) and cultured in minimum essential medium *α* medium (MEM-*α*, Gibco/Invitrogen, Carlsbad, Grand Island, NY, USA), supplemented with 10% fetal bovine serum (Gibco) at 37°C in a humidified atmosphere containing 5% CO_2_. The culture medium was changed twice per week. The basic characterization of UCB-MSCs is summarized in Supplementary Table 1. Primary BM-MSCs and human AT-MSCs were purchased from Promega (Gibco/Invitrogen, Heidelberg, Germany). For growth kinetics, the trypan blue exclusion method was performed to analyze the expansion of live cells. At each passage (P), MSCs were cultured for 5 days, then reseeded at a cell density of 2,000 cells/cm^2^. The PD at each passage was calculated by dividing the logarithm of 2 [20]. The analysis of PD was continued until the proliferation of cells was stopped.

### 2.2. In Multilineage Differentiation Potential

To assess multilineage potential, cells were incubated under specific conditions to induce differentiation into osteocytes, chondrocytes, and adipocytes. After differentiation, the multilineage potential was evaluated as previously described [21, 22]. Briefly, osteocyte formation was assessed by measuring the level of ALP staining (Sigma-Aldrich, St. Louis, MO, USA); chondrocyte formation was determined by safranin O staining (Sigma); adipocyte formation was assessed based on the staining of accumulated lipid vacuoles with oil red O (Sigma).

### 2.3. Isolation by Cell Size

MSCs were separated into three groups based on a diameter of 8 *μ*m as follows: nonsieved population (heterogeneous), population > 8 *μ*m (large), and population ≤ 8 *μ*m (small). Size-sieved samples were processed by methods summarized Supplementary Figure 1a. For isolation based on size, we prepared the filter considering the risk of damaging MSCs and safety using the Xiaogan Yaguang's filtration membrane tube (8 *μ*m pore size, Xiaogan, Hubei Province, China). First, the filtration membrane tube was inserted into a 50 mL culture tube. Next, 1 × 10^5^ MSCs/mL were loaded on a filtration membraned tube. Finally, the tube was centrifuged 1200 rpm for 5 min to obtain three populations (heterogeneous: unsieved; large: upper layer of filter; and small: lower layer of filter). For size measurements, cells were harvested, pelleted, suspended in media, and pipetted in a hemocytometer. Images were then acquired at 100x magnification (Nikon Instruments Inc. ECLIPSE TS100, Melville, NY, USA) in multiple regions. At the photographed regions, the sizes of live cells were analyzed using SABIA (MeTooSoft, Seoul, Korea). The light-scattering properties of the cells were measured using a flow cytometer (BD Biosciences, San Diego, CA, USA) as the probing beam. The forward scattering distribution histograms for each cell population were generated on a computer from raw data files of flow cytometry.

### 2.4. Cell Adhesion Assay

Cell adhesion assays were performed using the IncuCyte (Essen Bioscience, Ann Arbor, MI). Cells were seeded in three replicates at 2,000 cells/cm^2^ in 6-well flat dishes and grown at 37°C, with 5% CO_2_. Images were acquired at 6, 12, 18, and 24 hours using the automated image acquisition software. Cell numbers at each time point were also determined using the Cell Counter plugin in ImageJ [23].

### 2.5. Cell Surface Antibody Screening with Lysoplates

To screen the human surface marker of MSCs, 242 antibodies were lyophilized in 96-well plates (BD LysoplatesTM; BD Biosciences) at 0.5 *μ*g/well and incubated with 500,000 MSCs per well. With 20 min reconstitution on ice, the washed cells were stained with an Alexa Fluor® 647-conjugated goat-anti-mouse IgG secondary antibody (Molecular Probes, Eugene, OR). Flow cytometry was performed to measure the surface markers using a FACSCalibur instrument (BD Biosciences). The data from flow cytometry were analyzed in Excel 2013 (Microsoft, Redmond, WA) to generate heat maps [20].

### 2.6. Flow Cytometry and Sorting

To assess and analyze the surface marker on MSCs, cells were stained with human CD14, CD45, CD49b, CD49d, and HLA-DR (BD Biosciences)-fluorescein isothiocyanate (FITC) antibodies, human CD29, CD44, CD90, CD340, EGFR, HLA-ABC (BD Biosciences), and CD105 (Serotec, Kidlington, UK)-phycoerythrin (PE) antibodies, and human CD49f (BD Biosciences)-Alexa 647 antibody. Isotype controls were matched to the mouse to detect the nonspecific background signal as negative controls. The stained MSCs were determined with a FACSCalibur instrument. To sort using specific markers, MSCs were stained with an EGFR or CD49f monoclonal antibody. Both EGFR and CD49f were sorted to 95% purity using a FACSVantage cell sorting system (BD Biosciences).

### 2.7. Senescence-Associated *β*-Gal Staining (SA *β*-Gal Staining)

To assess the senescence in MSCs, SA *β*-gal staining was performed using a histochemical staining kit (Cell Signaling Technology, Danvers, MA, USA) according to the manufacturer's instructions. The percentage of senescent cells = the number of positively stained cells/total number of cells [20].

### 2.8. Western Blotting

Cells were lysed with RIPA buffer to extract proteins. A total of 10 *μ*g of each protein extract was electrophoresed on a sodium dodecyl sulfate-(SDS)-polyacrylamide gel, and then the resolved proteins were transferred to a nitrocellulose membrane. Blocked membranes were incubated with primary antibodies against phospho-p53 (pho-p53), p16, phospho-Rb (pho-Rb), p21, p53, Rb (Cell Signaling), and p16 (Abcam, Cambridge, UK), followed by horseradish peroxidase-conjugated secondary antibodies. Chemiluminescent intensity of immunoblotted bands was visualized using a ChemiDoc Imaging System (Bio-Rad, Hercules, CA, USA). The intensity of each band was normalized to that of *β*-actin (Novus Biologicals, Centennial, CO, USA).

### 2.9. Quantitative Real-Time PCR and Small Interfering RNA Experiments

Quantitative real-time PCR (qPCR) was performed with universally designed Oct4 or Nanog TaqMan probes using a LightCyclerTM 480 (Roche, Mannheim, Germany). The relative expression levels of Oct4 or Nanog mRNAs were normalized to *β*-actin mRNA expression. EGFR, CD49f, and control small interfering RNA (siRNA) were purchased from Dharmacon (Chicago, IL, USA). siRNAs for EGFR siRNA, CD49f siRNA, or scrambled siRNA were transfected for 24 h using DharmaFECT Reagent (Dharmacon) according to the manufacturer's instructions. The four different siRNA duplexes were described on Supplementary Table 2. When cells were examined at multiple passages, de novo transfection of siRNAs was performed at each passage.

### 2.10. Animal Model of Emphysema

All animal experiments were reviewed and approved by the Institutional Animal Care and Use Committee of MEDIPOST Co., Ltd. (MP-2015-6-5). C57BL/6 mice were purchased from Samtako BioKorea Co. Ltd. (Osan, Korea). To generate the elastase-induced model, 6-week-old female C57BL/6J mice were intratracheally instilled with porcine pancreatic elastase (0.4 U per mouse) (Sigma). The mice were then intravenously injected with 1 × 10^4^ of UCB-MSCs at day 7. After 7 days, all lung tissue preparation procedures were performed only for surviving animals. For histopathological evaluation, the lungs were perfused with phosphate-buffered saline (PBS) through the right ventricle and inflated with PBS through the trachea. The trachea was then ligated, and the lungs were removed and immersed in the same fixative overnight at room temperature. Fixed lungs were embedded in paraffin and sectioned to 4 *μ*m thickness. Sections from the paraffin blocks were assayed based on six nonoverlapping random fields per section stained with hematoxylin and eosin (H&E). The level of alveolarization was determined by measuring the mean linear intercept (MLI). The mean interalveolar distance was measured as the MLI, by dividing the total length of lines drawn across the lung section by the number of intercepts encountered, as described [24]. For immunofluorescence, lung sections were then incubated at 4°C overnight with primary antibodies including mouse anti-surfactant protein C (SP-C, Abcam), mouse anti-von Willebrand factor (vWF, Cell signaling), and human anti-*β*2-microglobulin (*β*2MG, Santa Cruz Biotechnology, Dallas, TX, USA), which were visualized using FITC or Cy3-labeled secondary antibody (Jackson ImmunoResearch Europe Ltd., Newmarket, UK). Nuclei were counterstained with 4′6-diamino-2-phenylindoled (DAPI, Sigma).

### 2.11. Statistical Analysis

Statistical analysis was performed with SPSS 18 (SPSS Inc., Chicago, IL, USA) using one-way analysis of variance followed by the least-significant difference (LSD) post hoc test. The data represented as mean ± standard deviation (SD) of values obtained in experiments performed at least in triplicate. *p* < 0.05 was considered to indicate statistical significance.

## 3. Results

### 3.1. UCB-MSCs Display a Heterogeneous Cell Size

**Figure 1:**
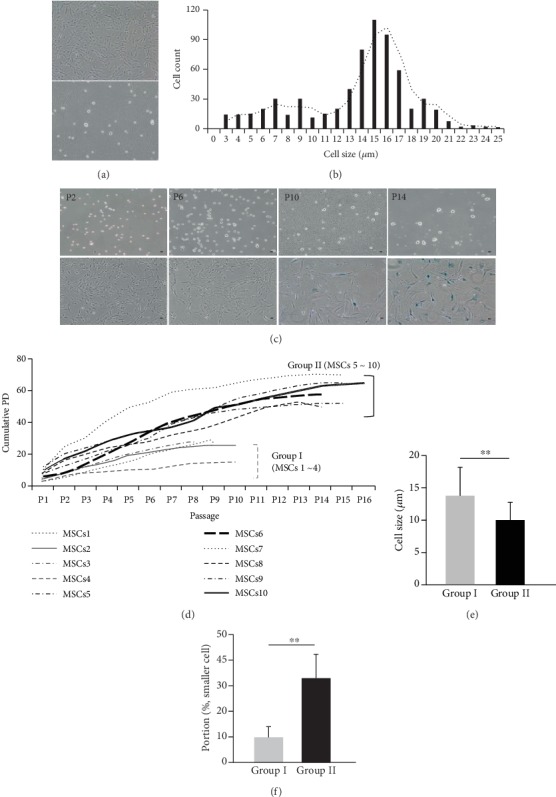
Cell size distribution of UCB-MSCs. (a) UCB-MSCs were observed as spindle-like fibroblastoid cells with heterogeneity regarding shape (upper panel) and single cell (lower panel) at P5. (b) The distribution of cell size was analyzed. (c) Cell size was increased in single cells following expansion. The cells were stained with SA *β*-gal. (d) Based on cell growth, 10 lots of UCB-MSCs were divided into two groups showing significant differences in cumulative PD, group I (UCB#1 to #4) and group II (UCB#5 to #10). (e) Cell size and (f) the proportion of smaller cells were measured in group I and group II at early stage (P2) (mean ± SD, ^∗∗^*p* < 0.01 compared to group I). Scale bar = 10 *μ*m; PD: population doubling.

UCB-MSCs expansion is dependent on adherence to plastic flasks, which is of concern regarding heterogeneity. Cell morphology was observed with a microscope, and single cells were obtained by trypsinization. UCB-MSCs were fibroblastoid morphology with heterogeneity regarding shape and size at P5 (Figure 1(a)). We analyzed the cell size of UCB-MSCs based on cell diameter. As expected, UCB-MSCs exhibited different cell sizes, ranging from 3 *μ*m to 25 *μ*m in diameter (Figure 1(b)). Moreover, with prolonged passaging *in vitro*, UCB-MSCs showed an increase in cell size, became morphologically enlarged and flattened, and SA *β*-gal activity, known as a marker of replicative senescence (Figure 1(c)). These results revealed that the cell size of UCB-MSCs is related to cellular senescence. Previous research reported that cells with a diameter ≤ 7~10 *μ*m (smaller cell or smaller size) are recycling stems cells, which rapidly proliferate, compared to that in other cell populations [14, 15]. In this study, small cells were ≤ 8 *μ*m in diameter. To further confirm the association between smaller size and the growth ability of UCB-MSCs, we analyzed the cumulative population doubling (PD) and cell size of UCB-MSCs from 10 different donors. The cellular expansion growth kinetics were evaluated by counting cells at each passage. We measured cell size based on images of single cells from four fields at the early stage and analyzed the proportion of smaller cells (Supplementary Table 3) in long-term culture. Next, we categorized these into two groups. Particularly, MSCs of group I stopped growing sooner and showed a faster senescence process with a lower cumulative PD than those in group II (group I, 24.0 ± 6.2 versus group II, 59.7 ± 7.8; *p* < 0.01; Figure 1(d) and Supplementary Figure 2). Interestingly, these groups showed significant differences in cell size (group I, 13.6 ± 5% versus group II, 10.1 ± 5%; *p* < 0.01; Figure 1(e)) and the portion of smaller cells (group I, 37.7.5 ± 5% versus group II, 15.5 ± 5%; *p* < 0.01; Figure 1(f)) at P2. Collectively, these results suggest that cell size is related to heterogeneity regarding the cell growth and senescence of UCB-MSCs.

### 3.2. The Small Cell Population of UCB-MSCs Possesses Enhanced Stem Cell Properties

**Figure 2:**
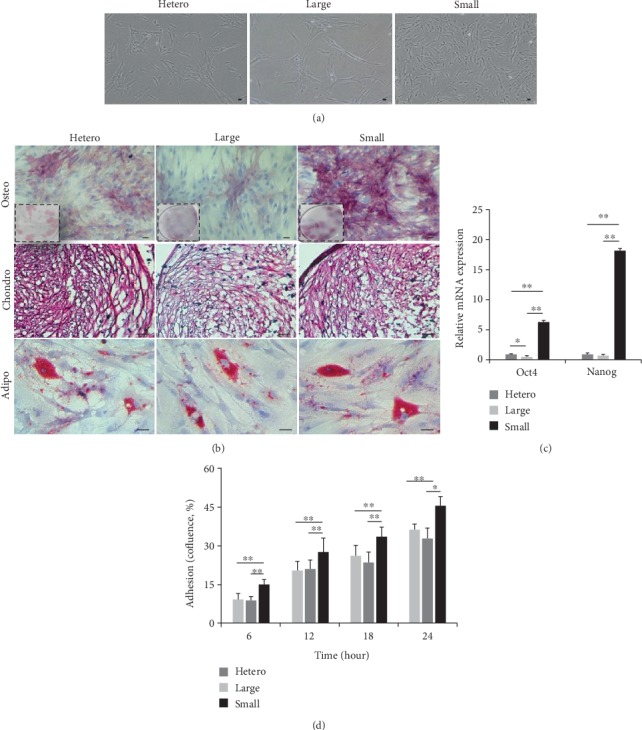
Stem cell characterization of differentially-sized populations. (a) Cell morphology was analyzed by microscopy (scale bar = 10 *μ*m). Three populations were observed with spindle-shape morphology. (b) During incubation in specialized induction media, multilineage differentiation was evaluated by staining for typical lineage markers. Among the three populations, osteogenic cells were analyzed based on bone-type ALP (upper panel). The picture with full range is shown in the boxed insert. Chondrogenic cells accumulated sulfated proteoglycan that stained positively for safranin O (middle panel). Adipogenic cells showed enhanced lipid vacuoles within the cytoplasm, which were stained with oil red O (lower panel; scale bar = 50 *μ*m). Nuclei were counterstained with hematoxylin. (c) Stemness genes (*Oct4*, *Nanog*) were quantified by qPCR. The expression levels of all genes were normalized to those of *β*-actin, and expression levels were normalized to those observed in heterogeneous cells, defined as 1-fold expression (mean ± SD, *n* = 3; ^∗^*p* < 0.05, ^∗∗^*p* < 0.01). (d) The confluence of the three cell populations was determined based on adherence to the culture flask at different culture times (mean ± SD, *n* = 3; ^∗^*p* < 0.05, ^∗∗^*p* < 0.01).

After isolating differentially sized populations (hetero, large, and small), we compared their stem cell properties including morphology, immunophenotype, differentiation ability, stemness, and adhesion potentials. A similar spindle-shaped morphology was observed in the three populations (Figure 2(a)). Immunophenotypic analysis revealed that the three populations were positive for the expression of CD29, CD44, CD90, CD105, and human HLA-ABC, but negative for CD14, CD34, CD45, or HLA-DR, according to the International Society Cell Therapy (ISCT) criteria [25] (Table 1). To investigate multilineage differentiation, cells were cultured in stimulation media and assessed by staining for alkaline phosphatase (ALP), safranin O, and oil red O, as positive markers for osteogenic, chondrogenic, and adipogenic differentiations, respectively (Figure 2(b)). A direct comparison of small cells to the other populations demonstrated similar differentiation potential into chondrogenic or adipogenic lineages. Notably, for osteoblasts, small cells expressed strong ALP activity and widespread staining, as compared to that in the other cells (Figure 2(b)). Regarding stemness-related genes (*Oct4*, *Nanog*), expression levels in small cells were significantly higher than those in the other populations (Figure 2(c)). To examine adhesion potential, we analyzed confluence during various culture times using IncuCyte. Data showed differences in the adherence of small cells to the culture flask compared to that with hetero or large cells (Figure 2(d)). Taken together, these results suggest that small-sized populations have enhanced properties such as stemness, adhesion, and osteogenic lineage differentiations.

### 3.3. The Small Cell Population Has Higher Growth Potential and a Lower Rate of Senescence

**Figure 3:**
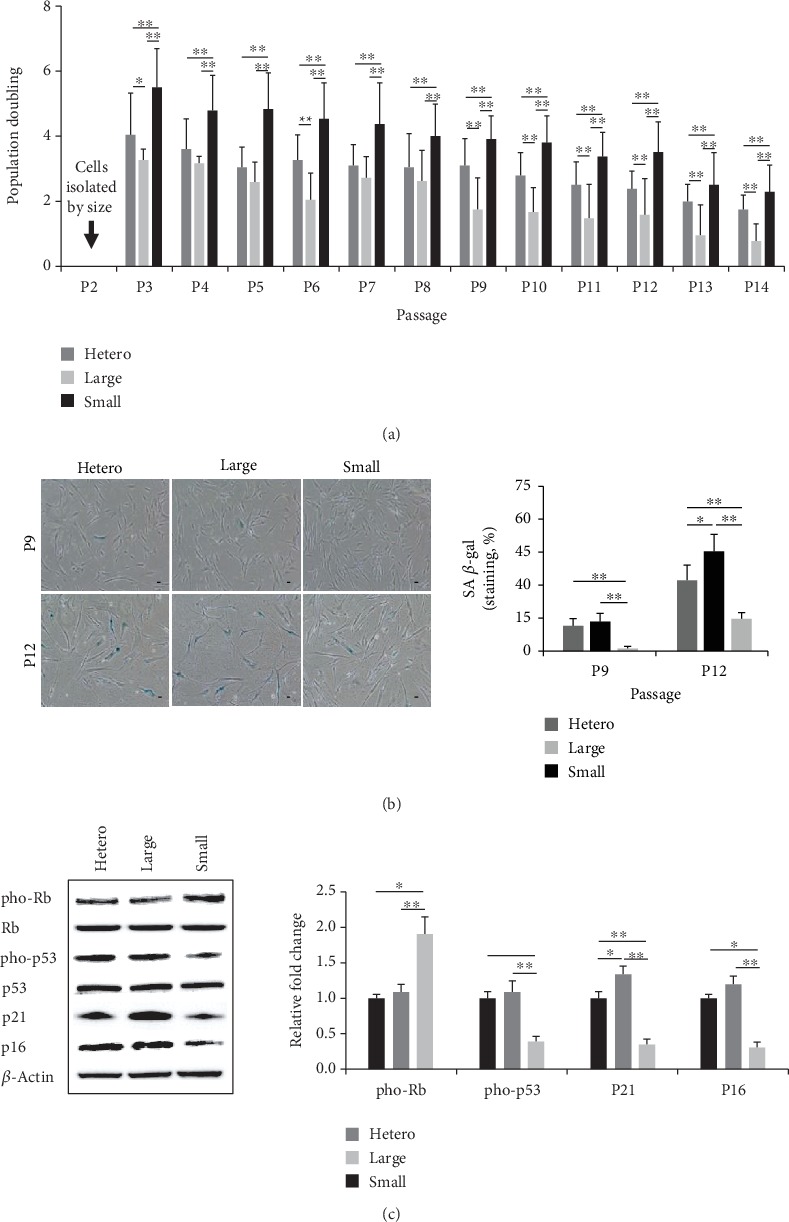
Proliferative ability and senescence of differently sized populations. (a) Growth kinetics of the three populations was assessed by monitoring PD until cell proliferation ceased. Black arrows indicate the point of isolation by size (mean ± SD, *n* = 4; ^∗^*p* < 0.05, ^∗∗^*p* < 0.01). (b) The senescent stage was evident based on positive SA *β*-gal staining in P9 and P12. Staining was quantified by positive cell counts. Error bars represent the means ± SD, *n* = 4; ^∗^*p* < 0.05, ^∗∗^*p* < 0.01. Scale bar = 10 *μ*m. (c) Expression of senescence-associated proteins (pho-p53, pho-Rb, p21, and p16) was measured at P12 by immunoblotting analysis. Expression levels were normalized to *β*-actin levels, with the expression levels in the heterogeneous group defined as 1-fold (mean ± SD, *n* = 3; ^∗^*p* < 0.05, ^∗∗^*p* < 0.01); PD: population doubling.

For therapeutic use, higher proliferative potential and decreased senescence are important parameters. Here, we investigated the growth kinetics and cellular senescence of three differently sized populations. All cells were continuously passaged in culture flasks at regular intervals until growth ceased. PD was measured for every passage MSC from four different donors. In culture, small cells exhibited significantly greater expansion capacity, whereas large cells showed the lowest growth rate at all passages (Figure 3(a)). To determine whether various features of cellular senescence were similar among the three populations, we tested SA *β*-gal staining and senescence-related protein expression. SA *β*-gal staining revealed no positive cells in the small-sized populations, whereas the mean in heterogeneous and large cells was 11.7 ± 2.8% to 16.3 ± 3.8% by P9. Further, SA *β*-gal expression dramatically increased in all populations to P12, but the level in small cells was significantly lower than that in the other groups (Figure 3(b)). Because cell cycle regulators are associated with cell senescence, we assessed pho-p53, pho-Rb, p21, and p16 by immunoblotting. The expression of pho-p53, p16, and p21 was lower in small cells, whereas the level of pho-Rb was increased, compared to those in heterogeneous and large cells at P12 (Figure 3(c)). Thus, the small-sized population exhibited the highest growth potential and lowest senescence.

### 3.4. Both EGFR and CD49f Mediate Various Characteristics of Small Cells

**Figure 4:**
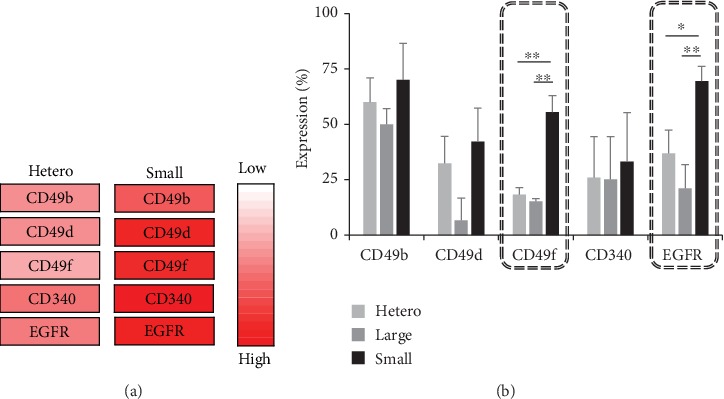
Screening for cell surface markers in UCB-MSCs with altered expression based on cell size. (a) Heat map analysis showing upregulated cell surface antigens in small cells compared to levels in heterogeneous cells. (b) To confirm the increase in surface proteins observed during screening, the protein expression of CD49b, CD49d, CD49f, CD340, and EGFR was assessed by flow cytometry using cells from three different donors (means ± SD, *n* =  3; ^∗^*p* < 0.05, ^∗∗^*p* < 0.01). Both CD49f and EGFR showed significant increases in small cells compared to expression in other cells (gray box).

A variety of surface proteins has been proposed to govern MSC features such as stemness and differentiation potential. Thus, we hypothesized that small cells might employ cell surface proteins to actively control stem cell properties. To test this hypothesis, we utilized a surface marker array containing antibodies against 242 CD markers to screen for expression differences between heterogeneous or small cells (Supplementary Table 4). As a result, we identified five cell surface proteins that were markedly upregulated in small cells, including CD49b, CD49d, CD49f, CD340, and EFGR (Figure 4(a)). To further examine these screening results, we measured the expression levels of these five surface proteins from three different donors by flow cytometry. The data shown in Figure 4(b) confirmed that EGFR and CD49f were significantly upregulated in small cells compared to levels in heterogeneous or large cells. Furthermore, the expression of EGFR or CD49 dramatically decreased after passaging. In detail, the expressions of EGFR^+^CD49^+^ on small cell were dramatically decreased from 70% at passage 3 into 40% at passage 7 (Supplementary Table 5). To verify the role of EGFR or CD49f in stem cell properties, we silenced them using siRNA in small cells (Supplementary Figure 3).

**Figure 5:**
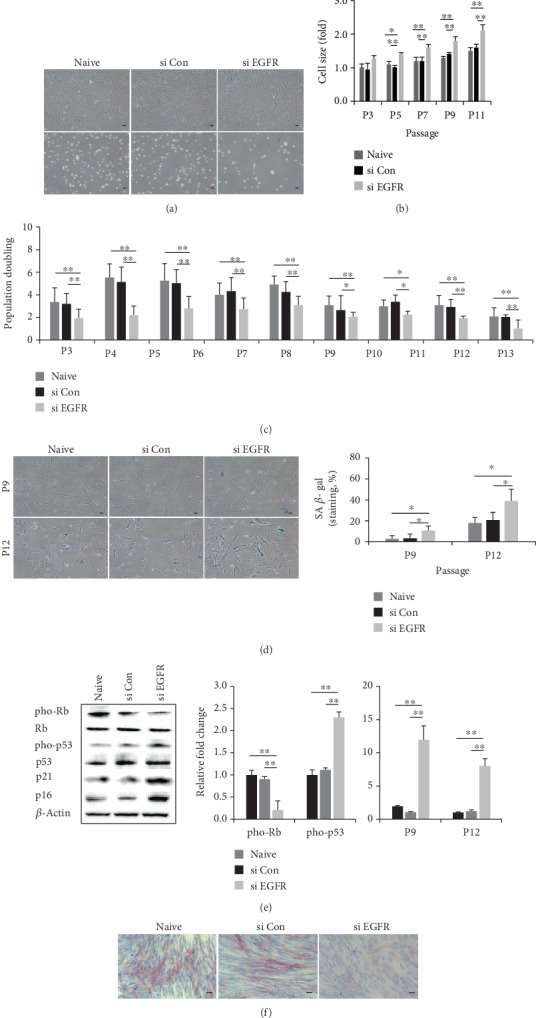
EGFR knockdown induces senescence and results in a loss of differentiation capability. Small-size populations of mesenchymal stem cells (MSCs) were transfected with scramble siRNA (si Con) or EGFR siRNA (si EGFR). (a) Morphological differences were observed by microscopy for adhesion (upper panel) or single cells (lower panel, scale bar = 10 *μ*m). (b) Cell sizes of the three populations were assessed during expansion. The expression levels were normalized to those observed in naïve cells, defined as 1-fold expression (mean ± SD, *n* = 45; ^∗^*p* < 0.05, ^∗∗^*p* < 0.01). (c) Cells were cultured under regular conditions and population doubling (PD) was assessed until cell growth ceased (means ± SD, *n* = 3; ^∗^*p* < 0.05, ^∗∗^*p* < 0.01). (d) The cells were stained with SA *β*-gal, and the activity was measured by counting positively-stained cells at P9 or P12 (means ± SD, *n* = 3; ^∗^*p* < 0.05, scale bar = 10 *μ*m). (e) The expression of senescence-associated proteins was measured by immunoblotting analysis and *β*-actin was used as a control at P12. The expression levels were normalized to those observed in naïve cells, defined as 1-fold expression (means ± SD, *n* = 3; ^∗∗^*p* < 0.01). (f) After 2 weeks of osteogenic induction, the level of ALP was determined by staining (scale bar = 50 *μ*m).

**Figure 6:**
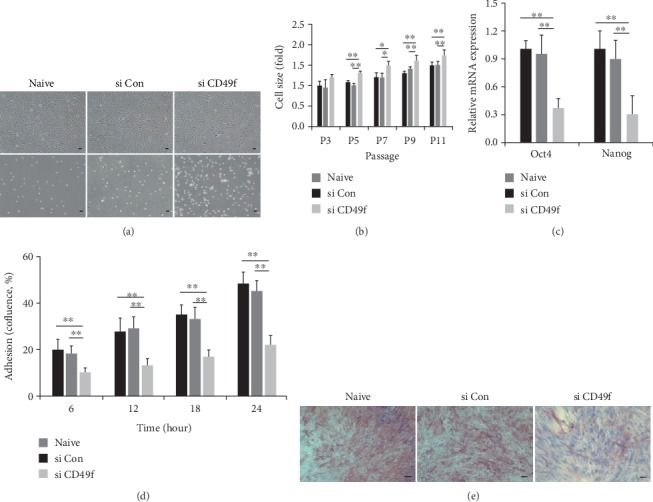
Silencing CD49f inhibits stem cell properties. Small cells were treated with scramble siRNA (si Con) or CD49f siRNA (si CD49f). (a) Morphological differences were observed by microscopy for adhesion (upper panel) or suspension cells (lower panel, scale bar = 10 *μ*m). (b) Cell sizes of the three populations were assessed during expansion. The expression levels were normalized to those observed in naïve cells, defined as 1-fold expression (mean ± SD, *n* = 70; ^∗^*p* < 0.05, ^∗∗^*p* < 0.01). (c) The stemness genes (*Oct4*, *Nanog*) were quantified by qPCR. The expression levels of all genes were normalized to those of *β*-actin, and levels were normalized to those observed in naïve cells, defined as 1-fold expression (mean ± SD, *n* = 3; ^∗∗^*p* < 0.01). (d) Adhesion potential was analyzed based on confluence in the culture flask for different culture times (mean ± SD, *n* = 3; ^∗∗^*p* < 0.01). (e) Osteogenic cells were measured by bone-type ALP staining (scale bar = 50 *μ*m).

Compared to those in scramble siRNA-transfected cells (si Con), cells from three different donors transfected with siRNA against EGFR or CD49f were assessed for morphology, cell size, growth rate, stemness, adhesion potential, and senescence phenotypes. First, in EGFR-silenced cells, larger morphology and increased cell size were observed (Figures 5(a) and 5(b)). Further, they exhibited significantly lower expansion capacity, whereas the control group (naïve, si Con) had a higher growth rate (Figure 5(c)). Based on the senescence phenotype, SA *β*-gal activity was significantly augmented in EGFR-silenced cells (Figure 5(d)), with a concomitant change in pho-p53, pho-Rb, p21, and p16 expression at P12 (Figure 5(e)). Moreover, EGFR-silenced cells showed restored osteogenic differentiation based on ALP staining (Figure 5(f)). In contrast, the stemness and adhesion potential were unaffected by EGFR siRNA (data not shown). Next, CD49f-silenced cells were flatter and showed an increase in size (Figures 6(a) and 6(b)). Stemness gene levels (*Oct4*, *Nanog*) in CD49f-silenced cells were significantly lower than those in the control groups (naïve, si Con, Figure 6(c). Greater adhesion potential was also observed compared to that in the control groups, and cells quickly adhered to culture flasks compared to that with CD49-silenced cells (Figure 6(d)). CD49f silencing also resulted in significantly reduced osteogenesis, as confirmed by ALP staining (Figure 6(e)). In contrast, cell growth and the senescence phenotype were not changed in the CD49 siRNA group (data not shown). To determine whether EGFR or CD49f expression is related to stem cell properties in small cells, we sorted cells based on EGFR or CD49f expression using an antibody. The sorted cells were purified by ≥95% (Supplementary Figure 4), and sorted groups were validated by evaluating ALP efficiency as an osteoblast marker. SA *β*-gal activity was analyzed as an indicator of senescence. We found that EGFR^+^ cells showed higher osteogenic potential and lower senescence than EGFR^−^ cells (Supplementary Figure 5a). Additionally, higher ALP activity was detected in CD49f^+^ cells than in CD49^−^ cells (Supplementary Figure 5b). Thus, these data indicate that the suppression of EGFR or CD49f affects markers that control the biological activity of small-sized populations, suggesting their utility as potential markers.

### 3.5. Effect of Small-Sized Cells in Different Adult Tissues

To investigate whether bone marrow-derived MSCs (BM-MSCs) or adipose tissue-derived MSCs (AT-MSCs) from different adult sources have small cell features, we analyzed their morphology and cell size. As shown in Supplementary Figure 6a, both BM- and AT-MSCs showed heterogeneity regarding shape and cell size. BM- and AT-MSCs exhibited different cell sizes and were larger compared to UCB-MSCs. UCB-MSCs were used as controls, and cells showed average diameters of 15.5 ± 3.5 *μ*m (BM), 17.7 ± 3.5 *μ*m (AT), and 11.5 ± 3.5 *μ*m (UCB) at P2 (Supplementary Figure 6b). An analysis of growth ability showed greater potential for small cells as compared to that of heterogeneous MSCs from the three sources. This increased growth rate with small cells was sustained long-term, at least until passage 6 (Supplementary Figure 6c). Next, we measured potency markers including CD49b, CD49d, CD49f, CD340, and EGFR from the three sources of MSCs by FACS. Interestingly, the data confirmed that EGFR and CD49f were significantly upregulated in UCB-MSCs compared to levels in BM- or AT-MSCs (Supplementary Figure 6d). Collectively, the small-sized population from BM- and AT-MSCs also possessed higher growth potential than heterogeneous cells.

### 3.6. Small Cells Enhance the Therapeutic Potential of UCB-MSCs in an Animal Model

**Figure 7:**
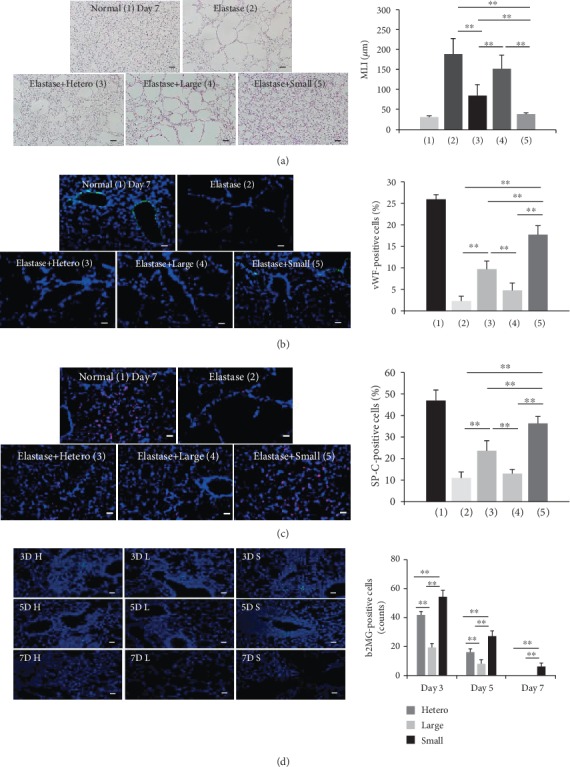
Improved therapeutic effect of the small-size population in an elastase-induced emphysema model. (a–c) Mice were exposed to elastase for 7 days and then intravascularly injected with cells of different sizes derived from UCB-MSCs. (a) The histological assessment at 7 days based on lung sections stained with H&E. Morphometric analysis quantified the mean linear intercept (MLI) (mean ± SD, *n* = 40; ^∗∗^*p* < 0.01). (b) Representative immunofluorescence photomicrographs of vWF staining in the lungs of each group. vWF was labeled with FITC (green) and nuclei were labeled with DAPI (blue). The number of vWF-positive cells was counted in six randomly chosen fields. The percentage of vWF-positive cells was normalized based on the number of nuclei (mean ± SD, *n* = 6; ^∗∗^*p* < 0.01). (c) SP-C was labeled with Cy3 (red) and nuclei were labeled with DAPI (blue). The number of SP-C-positive cells was counted in six randomly chosen fields. The percentage of SP-C-positive cells was quantified based on the number of nuclei (mean ± SD, *n* = 6; ^∗∗^*p* < 0.01). (d) The number of human *β*2MG-stained cells was analyzed from at least six randomly chosen fields at 3, 5, and 7 days after cell injection (mean ± SD, *n* = 5; ^∗∗^*p* < 0.01). Scale bars = 50 *μ*m. D: day; H: heterogeneous; L: large; S: small.

We then hypothesized that small MSCs could be employed for tissue regeneration. To compare the therapeutic potential of small-sized populations with that of heterogeneous or large cells for lung disease, we analyzed therapeutic outcomes with these populations using the elastase-induced emphysema mouse model. The animals were injected with elastase and intravenously injected with differently sized populations of UCB-MSCs (1 × 10^4^) at 7 days after elastase injection. In mouse lung tissue, morphometric analysis was performed by measuring the mean linear intercept (MLI), and angiogenesis was assessed based on von Willebrand factor (vWF) expression, whereas lung repair was analyzed by surfactant protein C (SP-C) levels at 7 days posttransplantation. The elastase-induced increase in MLI was significantly attenuated by treatment with heterogeneous or small cells, with the greatest reduction using small cells. Specifically, the alveolar phenotype with small cell treatment showed dramatic regeneration, and MLI returned to a near-normal range (70.9 ± 2.3 *μ*m, Figure 7(a)). The elastase-induced decrease in vWF or SP-C expression was significantly enhanced by the injection of heterogeneous or small cells, but not by large cells (Figures 7(b) and 7(c)). The expression of vWF and SP-C was higher with small cell treatment than with heterogeneous cells. For the engraftment of infused cells, we tested the number of engrafted cells by staining the lung tissue with an antibody specific to human *β*2MG (green) at 3, 5, and 7 days after injection, and small-sized MSCs resulted in greater engraftment capacity in lungs (Figure 7(d)). In a different set of experiments, we compared the therapeutic effect based on the proportion of small-size cells using the elastase-induced emphysema mouse model. For the two MSC lines used, with MSC-I, as the larger lot (group I of Figure 1(d)), the proportion of small cells was 9.2%. In the MSC-II group, as the smaller lot (group II of Figure 1(d)), the rate of small cells was 35%. The elastase-induced morphological changes and impaired angiogenesis were attenuated with the transplantation of these two MSC groups. The MLI was significantly lower in lungs injected with MSC-II than in lungs receiving MSC-I (Supplementary Figure 7a). In addition, the level of vWF in animals treated with MSC-II was significantly higher than that in mice treated with MSC-I (Supplementary Figure 7b). There have been several reports about paracrine effects on treatment of MSCs on various disease models, including emphysema [26–28]. Also, engrafted MSCs into the lung epithelium of acute respiratory distress syndrome have secreted paracrine factors, such as KGF (keratinocyte growth factor), VEGF (vascular endothelial growth factor), and HGF (hepatocyte growth factor), to promote the protective effects of pulmonary vascular permeability and the proliferation of epithelial cell [29]. Additionally, PGE2 (prostaglandin E2) secreted by MSCs suppressed the inflammatory cytokines and stimulated the alveolar macrophage to secrete the anti-inflammatory cytokines, IL-10 [27, 28]. To explain the therapeutic effects of small cells, we analyzed the protein expression levels with a fluorescent human antibody array to compare the protein expression between small and heterogenous cells. Among biologic processing (Supplementary Figure 8a), the result of secretion protein has been demonstrated that VEGF, EGF, TIMP-2 (tissue inhibitor of metalloproteinases 2), TSP-1 (thrombospondin-1), and Decorin secreted by small cells were increased, markedly (Supplementary Figure 8b). Collectively, our result suggests that small-sized populations augment beneficial outcomes of lung regeneration in a lung disease model.

## 4. Discussion

The simplicity of MSC culture comes with concerns regarding the heterogeneity of the resulting cell population, which is overpassaging until the enough number of MSCs is obtained for clinical use. This heterogeneity remains a major concern, not only for gaining a general understanding of the biological function through which MSCs maintain their stemness, growth potential, senescence, and undergo differentiation toward specific lineage features but also with respect to achieving better outcomes in cell-based therapy. To overcome this heterogeneity, we focused on cell size and assessed the association between growth rate and the size of MSCs. Here, depending on the proportion of small cells, UCB-MSCs exhibited heterogeneity in growth and senescence during subculture.

Although previous studies have suggested that the small-sized population of BM- or UC-MSCs possesses the capacity for proliferation, differentiation, and delayed senescence [15–17], isolation protocols for small cells were not fully established due to the good manufacturing practices requirement for clinical trials. In the present study, we applied an isolation strategy as an easy approach, which obtained small cells and was associated with an advantage in terms of safety issues and yield during size separation. Aseptic processing is one of the most significant factors for success of GMP safety. Here, we use filter and centrifugation in a closed system as a new strategy to successfully solve previous contamination issues. Furthermore, the effect of the small-sized population from UCB-MSCs on stem cell features had not previously been reported. Here, we provide the first demonstration that this population exhibits enhanced stem cell properties, as compared to heterogeneous and large-sized cell populations. Interestingly, we observed that small-sized cells from UCB-MSCs more extensively differentiated into osteoblasts, as assessed by increased ALP staining, consistent with previous reports on BM-MSCs. Plastic adherence is a well-defined feature of MSCs when maintained in basic culture conditions using culture flask. Our data showed that the small-sized population exhibited the best adhesion. Next, we investigated stemness including *Oct4* and *Nanog*, as the expression of these genes is needed to maintain differentiation potential and growth activity [30, 31]. However, the effects of cell size on these markers had not been characterized. In our study, levels of *Oct4* and *Nanog* were significantly enriched in the small-sized population compared to those in the heterogeneous or large-sized population.

The growth ability of the small-sized population was significantly higher, and these cells could be extended for longer periods in culture than other populations. For clinical applications, the ability of MSCs to rapidly propagate in culture and a cell number of 1 × 10^9^ cells are desirable [32], making the small-sized population a useful model in allogeneic settings. In addition, small-sized populations can expand for more than 15 passages with a normal karyotype (Supplementary Figure 9). After extensive culture expansion, cellular senescence can be a major obstacle; this is mainly described as growth arrest and the loss of differentiation ability in MSCs [20, 32, 33]. In this study, we demonstrated that senescence of the small-sized population was slower than that of the heterogeneous or large population, suggesting the superiority of small-sized cells. Further, SA *β*-gal staining revealed significantly reduced expression of this senescence marker in the small-sized population. Two major proteins, p53 and Rb, have been shown to be the main contributors to senescence. When cellular senescence progresses, p53 protein activates its transcriptional targets such as p21 [34]. Rb is maintained in its phosphorylated form during senescence and binds to E2F protein family members to suppress their transcriptional activity [35]. Our previous data demonstrated that the levels of pho-p53, p16, and p21 were significantly increased during expansion, whereas the level of pho-Rb was decreased [34]. These proteins were strongly expressed in heterogeneous and large cells, but their expression was markedly lower in small cells. In UC-MSCs, Majore et al. demonstrated that smaller-size populations exhibit low SA *β*-gal activity, suggesting that the small-size population might be precursors of mature and larger cells [17].

Currently, cell surface proteins are the most widely used makers [36, 37], not only as the minimal criteria to define MSCs but also as quality control markers to select functional MSCs. In addition, numerous studies have shown that these surface antigens control various biological functions of MSCs including gene expression [36, 37]. Here, we assessed cell surface marker expression in small-sized populations using FACS screening. We identified five proteins that were increased in small cells. Based on our results, the small size of UCB-MSCs is regulated by EGFR and CD49f, major mediators of therapeutic activation, which was confirmed by knockdown or sorting. First, epidermal growth factor (EGF) is a well-known growth factor/cytokine that binds the EGFR, and these mechanisms increase cell growth and differentiation without affecting pluripotency [38, 39]. As EGFR tends to decrease during aging, it is highly expressed in early cell types to regulate cellular senescence via phosphoinositide 3-kinase (PI3K) signaling, which is one of the major pathways downstream of EGFR [40–42]. Our results also showed that EGFR in small-sized cells is related to growth, differentiation, and senescence. Next, integrin *α*6 (CD49f) is a cell surface antigen that controls a variety of cellular activities. Of note, it was reported that CD49f enhances differentiation potential and maintains stemness via the direct regulation of Oct4 and Sox2 in spheroid-form MSCs [43]. Another report proposed that CD49f is a marker of early progenitor cells in cultured BM-MSCs; CD49f^high^ MSCs were found to be more clonogenic and differentiated than CD49f^low^ cells [44]. In embryonic stem cells, CD49f plays a predominant role in the initial attachment of cells to the ECM [45, 46]. We demonstrated that CD49f mediates stemness, cell adhesion, and differentiation in small-sized populations. Taken together, our data provide experimental evidence that both EGFR and CD49f are quality control markers and predict small size.

Interestingly, our results showed that UCB-MSCs express significantly higher levels of EGFR and CD49f compared to MSCs from the other two sources. Previous reports showed that CD49f levels are higher in UCB-MSCs than in BM-MSCs [47]. Several reports have proposed that neonatal tissues exhibit certain biological properties that differ from MSCs originating from adult sources [47, 48]. Thus, our findings indicated that UCB-MSCs from neonatal tissue are generally smaller than cells from adult tissues. It is also reported that fetal MSCs remain consistently small and are multipotent even after expansion [12].

Indeed, we first showed that small UCB-MSCs led to a greater improvement in beneficial effects by not only improving the engraftment capacity of infused stem cells but also by reducing lung damage in an emphysema mouse model. The intravascular delivery of stem cells has been the most popular route for cell-based therapy in clinical application [49]. MSC migration and engraftment to injury sites have been tested previously in several disease models [50–52]. However, the numbers of intravenously transplanted cells remain low, even though MSCs showed beneficial effects in an emphysema model [53]. It is often reported that large cells cause severe vascular obstruction during stroke in rats [54]. Moreover, Kim et al. showed that the distribution of injected AT-MSCs was only detected in the lung 1 day after intravenous injection [55]. Importantly, small-sized populations maintain higher cell numbers for longer periods compared to other groups. In this study, although heterogeneous cells provided partial and minimal protection against elastase-induced lung injury *in vivo*, small cells resulted in the greatest attenuation. For example, small cells promote functional lung regeneration by preventing impaired alveolarization and angiogenesis. However, the optimal dose for transplantation needs to be addressed for successful clinical trials. Most reports have suggested the therapeutic efficacy of different doses of BM- and AT-MSCs (1 × 10^5^ or 5 × 10^5^ cells) administered intravenously in an emphysema model [26, 56–58], whereas our results indicate that a dose of 1 × 10^4^ small cells is sufficient for therapeutic effects. Cumulatively, our findings suggest that the small-sized population is the most suitable for future clinical use. There have been several reports about paracrine effects on treatment of MSCs on various disease models, including emphysema [26–28]. Previous reports also supported that engrafted MSCs in the emphysema-secreted paracrine factors, such as VEGF, and EGF, to promote mechanism for the protective effects of pulmonary tissues from elastase injury [26, 59]. In our data, the antibody array results demonstrated that VEGF, EGF, TIMP-2, TSP-1, and Decorin secreted by small cells were increased, markedly. Recently, we have found that Decorin is also one of key factors on the immunomodulation of MSCs, related to repair the damaged lung by inhibiting the inflammatory reaction [60].

In this context, small-sized populations become larger during cell expansion during general monolayer culture. Therefore, maintaining this small size has become an important parameter for further cell-based therapy based on *in vitro* cultures of small populations. Recent reports have suggested that suspension culture is crucial to produce smaller MSCs. For example, compared to the monolayer culture of MSCs, various methods including spheroid formation, aggregates, and bioreactors have been developed to maintain the smaller size of MSCs, and these methods significantly enhance therapeutic potential in several disease models [61–63]. Further work is needed to determine whether small-sized populations based on suspension methods also have practical potential for cell-based therapy.

## 5. Conclusion

In conclusion, we provide evidence supporting small-sized culture as a contributor to the enhanced stem cell properties of UCB-MSCs. We further demonstrated that both EGFR and CD49f are new markers that regulate small-sized populations. Therefore, our study suggests an important role of small size in potentially improving the efficacy of MSC transplantation, which will advance new therapeutic modalities for the preparation of next-generation MSC-based therapies.

## Figures and Tables

**Table 1:** Surface marker expression among different stem cell populations.

Marker	CD14	CD34	CD45	HLA DR	CD29	CD44	CD90	CD105	HLA ABC
Heterogeneous	-	-	-	-	+	+	+	+	+
Large	-	-	-	-	+	+	+	+	+
Small	-	-	-	-	+	+	+	+	+

+: more than 95%; -: less than 5%.

## Data Availability

The datasets generated during the current study are available from the corresponding author on reasonable request.

## Supplementary Materials

Supplementary Materials Figure 1: differently sized populations of UCB-MSCs after sieving. Supplementary Figure 2: cumulative PD in group I (n = 4) and group II (n = 6) of 10 individual cases of UCB-MSCs. Supplementary Figure 3: silencing of EGFR or CD49f gene expression in UCB-MSCs. Supplementary Figure 4: EGFR and CD49f sorting by flow cytometry. Supplementary Figure 5: features of UCB-MSCs sorted based on EGFR or CD49f expression. Supplementary Figure 6: heterogeneous sizes of MSCs from different adult tissues. Supplementary Figure 7: improved therapeutic effect of small-size cell population on elastase-induced emphysema mouse model. Supplementary Figure 8: secretome analysis on MSCs. Supplementary Figure 9: karyotyping analysis of small-size population at passage 15. figure: basic characteristics of UCB-MSCs . Supplementary Table 2: cell size and proportion of smaller cells in UCB-MSCs from 10 different donors at P2.; Supplementary Table 3: Primer sequences used for indicated target genes and siRNA sequences. Supplementary Table 4: expression levels (%) of 242 human cell surface markers in UCB-MSCs at passage 5 in heterogeneous and small populations as analyzed by flow cytometry. Supplementary Table 5: the expression of EGFR and CD49f on small size cell during passaging as analyzed by flow cytometry.
