# Supplementary material for: A Small-Sized Population of Human Umbilical Cord Blood-Derived Mesenchymal Stem Cells Shows High Stemness Properties and Therapeutic Benefit
Source: Stem Cells Int. 2020 Apr 28;2020:5924983. doi: 10.1155/2020/5924983 (PMC7204153; doi:10.1155/2020/5924983)
Supplement: Supplementary Materials — Figure 1: differently sized populations of UCB-MSCs after sieving. Supplementary Figure 2: cumulative PD in group I (n = 4) and group II (n = 6) of 10 individual cases of UCB-MSCs. Supplementary Figure 3: silencing of EGFR or CD49f gene expression in UCB-MSCs. Supplementary Figure 4: EGFR and CD49f sorting by flow cytometry. Supplementary Figure 5: features of UCB-MSCs sorted based on EGFR or CD49f expression. Supplementary Figure 6: heterogeneous sizes of MSCs from different adult tissues. Supplementary Figure 7: improved therapeutic effect of small-size cell population on elastase-induced emphysema mouse model. Supplementary Figure 8: secretome analysis on MSCs. Supplementary Figure 9: karyotyping analysis of small-size population at passage 15. figure: basic characteristics of UCB-MSCs . Supplementary Table 2: cell size and proportion of smaller cells in UCB-MSCs from 10 different donors at P2.; Supplementary Table 3: Primer sequences used for indicated target genes and siRNA sequences. Supplementary Table 4: expression levels (%) of 242 human cell surface markers in UCB-MSCs at passage 5 in heterogeneous and small populations as analyzed by flow cytometry. Supplementary Table 5: the expression of EGFR and CD49f on small size cell during passaging as analyzed by flow cytometry. [file 5924983.f1.pdf]

Supplementary Materials

A small-sized population of human umbilical cord blood-derived mesenchymal stem cells shows high stemness properties and therapeutic benefit

Miyeon Kim, Yun Kyung Bae, Soyoun Um, Ji Hye Kwon, Gee-Hye Kim, Soo Jin Choi, Wonil Oh, Hye Jin Jin *

Biomedical Research Institute, MEDIPOST Co., Ltd., Seongnam 13494, Republic of Korea

[*]Corresponding authors:
Hye Jin Jin, Ph.D.
Biomedical Research Institute, MEDIPOST Co., Ltd., Seongnam, 13494, Republic of Korea
Tel: 82-2-3465-6780; Fax: 82-2-3465-6754; E-mail: genny77@medi-post.co.kr

Supplementary Methods

Human antibody array

To obtain superannuants small or heterogenous, cells were serum starved for 24 h, washed, and then cultivated for a further 24 h with fresh serum-free medium. The conditioned media from each sample was tested to a glass chip containing 507 antibodies (Human Antibody Array L series, RayBiotech, Peachtree Corners, GA) according to the manufacturer's protocol. The detail information of antibody array can be accessed in the webpage of RayBioteh (www.raybioteh.com/l-series-507-label-based-human-array-1-glass-slide-2). Fluorescence detection were analyzed using a GenePix 4000B scanner (Molecular Devices, Sunnyvale, CA), which were performed by e-biogen (Seoul, Korea) using GenePix Pro 6.0 software (Axon Instruments). Quantified values were normalized to the positive control. We compared to demonstrate the relative change in expression levels of each protein between group. (Small cell vs. Heterogenous cell).

Supplementary Figure 1. Differently-sized populations of UCB-MSCs after sieving. (a) Isolation methods by cell size. In three populations (heterogeneous, large, small), cells showed different cell sizes under the microscope (scale bar = 10 μm). (b) Cell sizes were confirmed by FSC analysis.

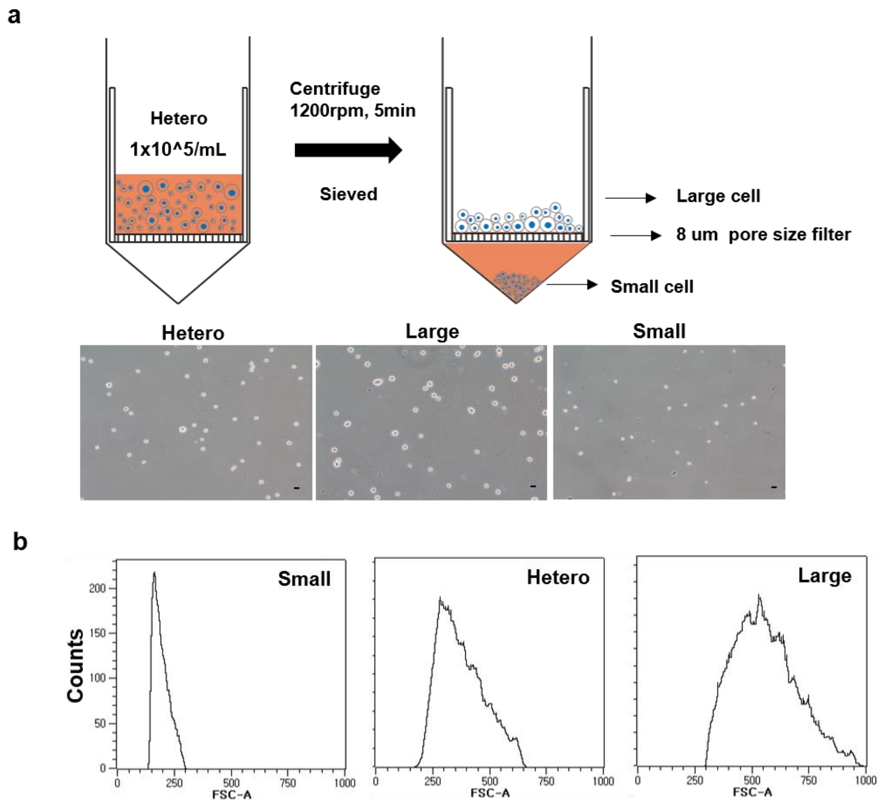

Supplementary Figure 2. Cumulative PD in group I (n = 4) and group II (n = 6) of 10 individual cases of UCB-MSCs (mean ± SD; ** p < 0.01).; PD, population doubling.

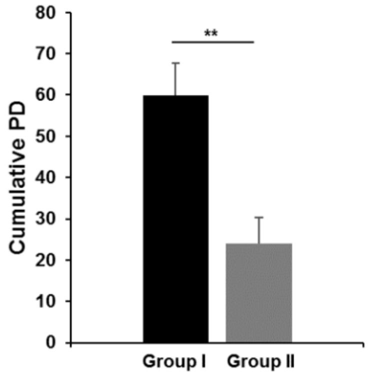

Supplementary Figure 3. Silencing of EGFR or CD49f gene expression in UCB-MSCs. Both *EGFR* and *CD49f* gene levels decreased in target siRNA-treated cells compared to those in naïve or siCon-treated cells. Downregulation of the two genes was confirmed by qPCR. The expression levels of all genes were normalized to those of *β-actin* in naïve cells, which was defined as 1-fold expression (mean ± SD, n = 3; ** $p < 0.01$).

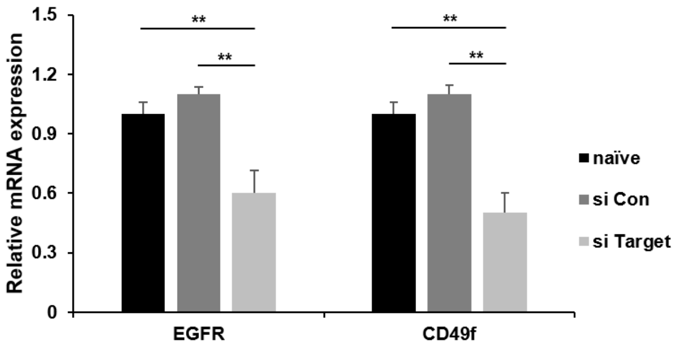

Supplementary Figure 4. EGFR and CD49f sorting by flow cytometry. The percentage of EGFR^{+} (a) or CD49f^{+} (b) cells was analyzed.

a

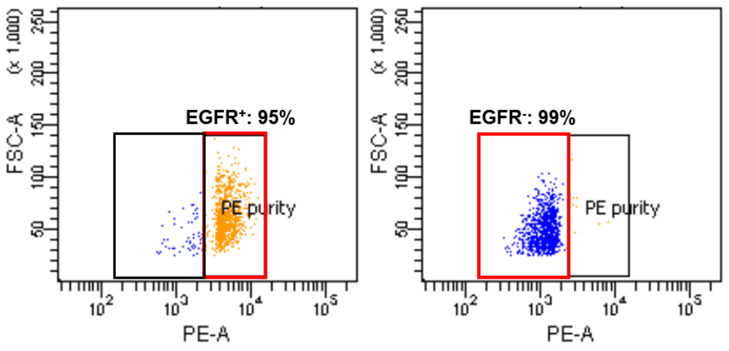

b

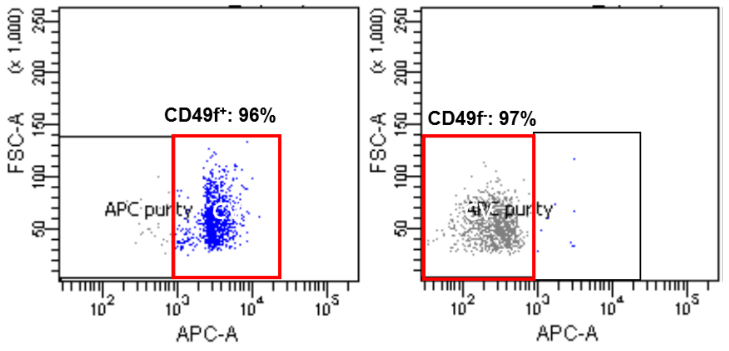

Supplementary Figure 5. Features of UCB-MSCs sorted based on EGFR or CD49f expression. (a)EGFR$^+$ and EGFR$^-$ cells were isolated using a FACSVantage sorting system. In each population, cell size, osteogenic features, and senescence were examined by microscopy, ALP staining, and SA β-gal staining, as indicated. CD49f$^+$ and CD49f$^-$ cells were isolated using a FACSVantage sorting system. In each population, cell size or osteogenic features was examined by microscopy and ALP staining. Scale bars = 50 μm.

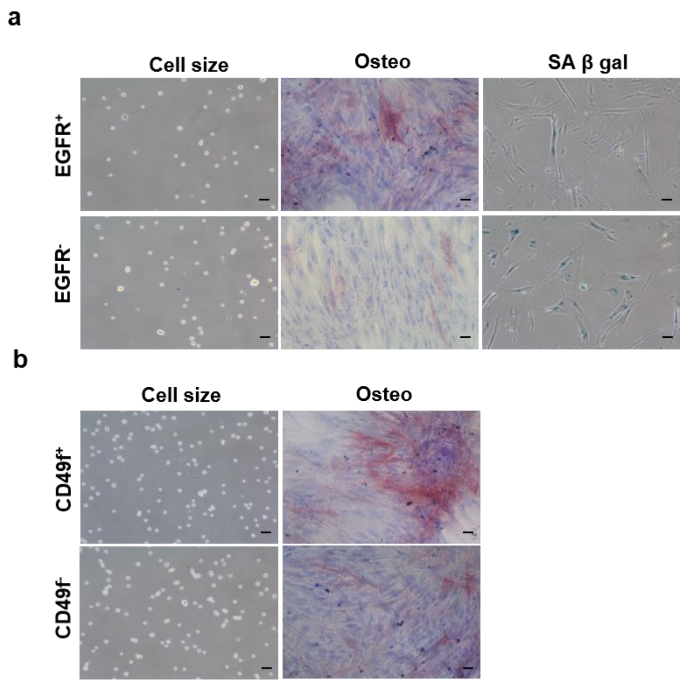

Supplementary Figure 6. Heterogeneous sizes of MSCs from different adult tissues. BM- and AT-MSCs were prepared. UCB-MSCs were used as a control. (a) MSCs from three sources were observed as spindle-like fibroblastoid cells with heterogeneous shapes and cell sizes (Scale bar = 10 μm). (b) The distribution of cell size was analyzed. (c) Growth kinetics were measured based on population doubling (PD) until P6 (mean ± SD, n = 6; ** $p < 0.01$). P: passage. (d) CD49b, CD49d, CD49f, CD340, and EGFR expression levels were checked by flow cytometry in MSCs from three sources (means ± SD, n = 3; ** $p < 0.01$). Expression levels were defined as 1-fold in UCB-MSCs. Both CD49f and EGFR showed significant increases in small cells compared to levels in other groups (gray box).

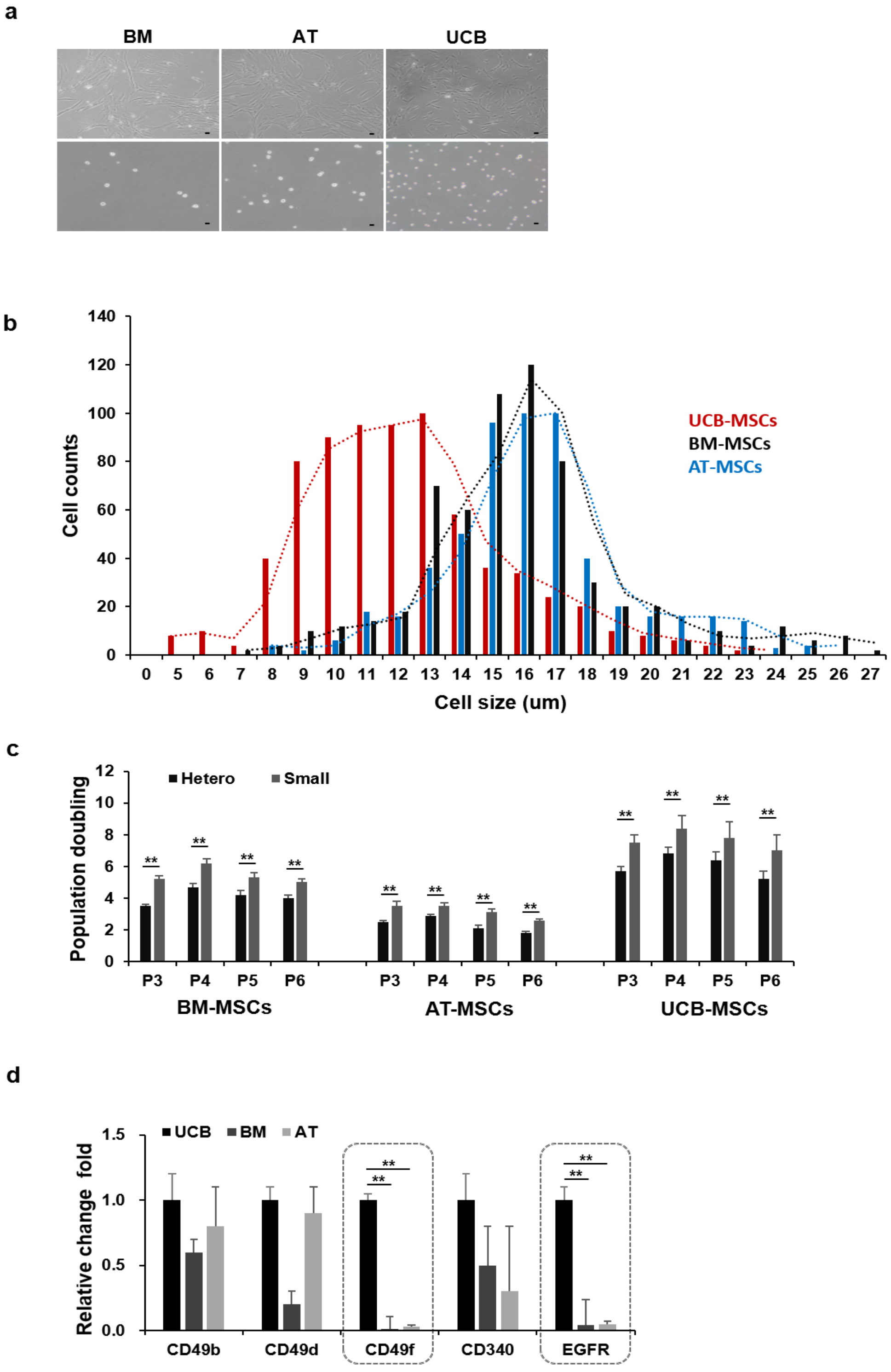

Supplementary Figure 7. Improved therapeutic effect of small-size cell population on elastase-induced emphysema mouse model. The two MSC lines were prepared as follows: MSC-I defined as the larger lot and MSC-II as the smaller lot. (a) Histological assessment of lung sections stained by H&E (scale bar = 50 μm). Morphometric analysis to quantify the MLI (mean ± SD, n = 40; ** p < 0.01). (b) Representative immunofluorescence photomicrographs of vWF staining in the lungs in each group. vWF was labeled with FITC (green), and nuclei were labeled with DAPI (blue). The number of vWF-positive cells was counted in six randomly chosen fields. Percentage of vWF-positive cells was quantified based on the number of nuclei (mean ± SD, n = 6; ** p < 0.01). Scale bar = 50 μm.

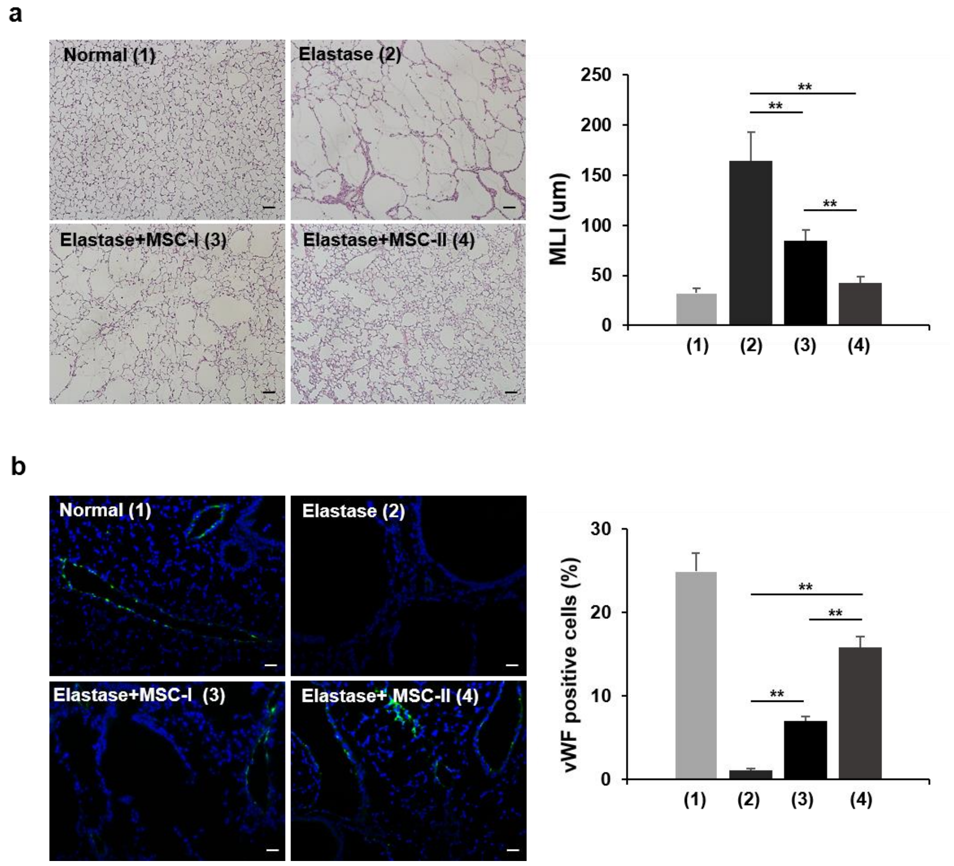

Supplementary Figure 8. Secretome analysis on MSCs. Biotin label-based antibody array was performed using the supernatants collected from small and heterogenous cells at passage 5. (a) Functional categories were classified by biological process annotation. (b) Quantification of the optical intensity of secretion factor. Intensity analysis showed up-regulated protein secretion in the small cell compared to in hetero cell. Protein levels were evaluated as the fold-increase, with data normalized to intensity of hetero, which was defined as 1. The quantification of optical density was performed and normalized to the positive control. VEGF, vascular endothelial growth factor; EGF, epidermal Growth Factor; TIMP-2, tissue inhibitor of metalloproteinases 2; TSP-1, thrombospondin-1.

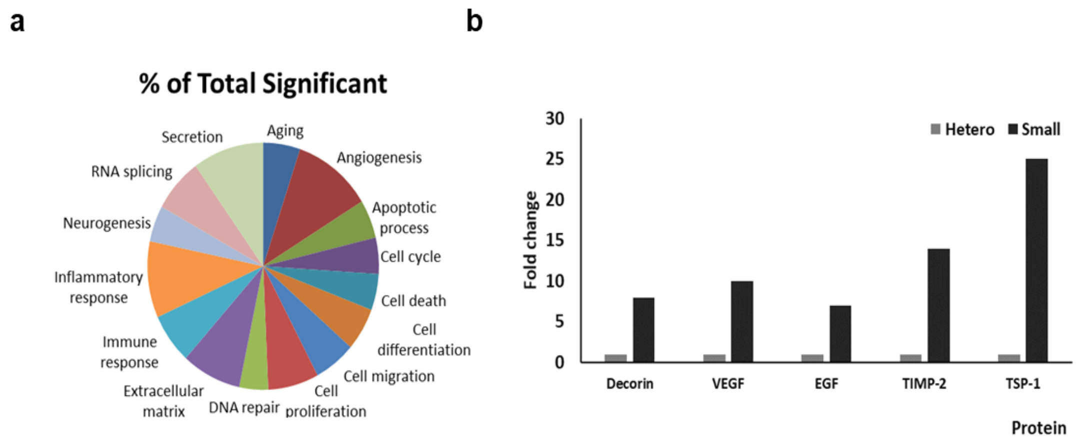

Supplementary Figure 9. Karyotyping analysis of small-size population at passage 15.

Normal Karyotype

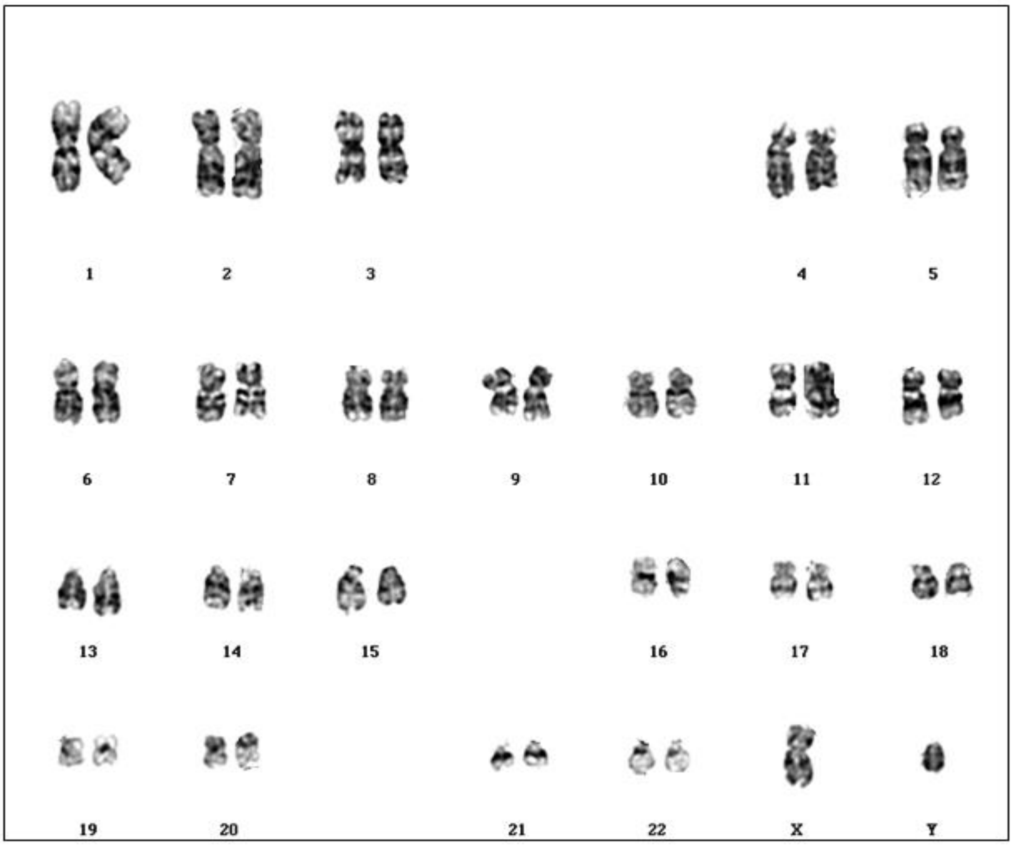

NAME: Small size cell, P15

RESULT: 46,XY (20)

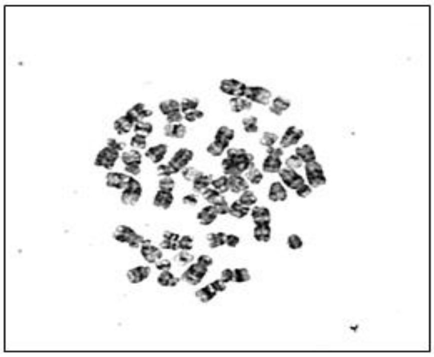

Supplementary Table 1. Basic characteristics of UCB-MSCs.

UCB-MSCs	Cell surface marker		Differentiation
	Positive	Negative	
#1	Pass	Pass	Pass
#2	Pass	Pass	Pass
#3	Pass	Pass	Pass
#4	Pass	Pass	Pass
#5	Pass	Pass	Pass
#6	Pass	Pass	Pass
#7	Pass	Pass	Pass
#8	Pass	Pass	Pass
#9	Pass	Pass	Pass
#10	Pass	Pass	Pass

UCBs were harvested from 10 independent donors (UCB #1 to 10). MSC features were analyzed by representative MSC marker expression or their differentiation capacity (positive: CD29, CD73, CD90, CD105, CD166 ≥ 80%; negative: CD14, CD45 ≤ 1.0%; differentiation: osteogenic, chondrogenic, adipogenic).

Supplementary Table 2. Cell size and proportion of smaller cells in UCB-MSCs from 10 different donors at P2.

	Group I				Group II					
Cells	MSCs1	MSCs2	MSCs3	MSCs4	MSCs5	MSCs6	MSCs7	MSCs8	MSCs9	MSCs 10
Small portion (%)	6.7	9.2	8.1	15.6	30.3	28.5	20	31.8	35	50
Cell size (average ± SD, μm)	12.9 ±4.3	13.8 ±4.7	14 ±4.2	14 ±5.4	9.8 ±2.8	10.9 ±3.8	11.1 ±2.8	10.2 ±2.9	9.6 ±2.5	9.3 ±2.6
Cell (number)	45	97	110	45	67	56	50	170	171	51

Smaller portion: percentage of MSCs ≤ 8 um in diameter. Data are shown as mean ± SD.

Supplementary Table 3. Primer sequences used for indicated target genes and siRNA sequences.

Gene	Primer Sequence (5′-3′)
Oct4	CAATTTGCCAAGCTCCTGA
	CGTTTGGCTGAATACCTTCC
Nanog	AGATGCCTCACACGGAGACT
	TTTGCGACACTCTTCTCTGC
EGFR	TTCCTCCCAGTGCCTGAA
	GGGTTCAGAGGCTGATTGTG
CD49f	TTTGAAGATGGGCCTTATGAA
	CCCTGAGTCCAAAGAAAAACC
β-actin	AGATGCCTCACACGGAGACT
	TTTGCGACACTCTTCTCTGC
Scramble siRNA	UGGUUUACAUGUCGACUAA
	UGGUUUACAUGUUGUGUGA
	UGGUUUACAUGUUUUCUGA
	UGGUUUACAUGUUUUCCUA
EGFR siRNA	CAAAGUGUGUAACGGAAUA
	CCAUAAAUGCUACGAAUAU
	GUAACAAGCUCACGCAGUU
	CAGAGGAUGUUCAAUAACU
CD49 siRNA	GGAUCGAGUUUGAUAACGA
	GGAUAUGCCUCCAGGUUAA
	GAAAGGGAUUGUUCGUGUA
	ACAGAUAGAUGAUAACAGA

Supplementalry Table 4. Expression levels (%) of 242 human cell surface markers in UCB-MSCs at passage 5 in heterogeneous and small populations as analyzed by flow cytometry.

Plate 1			Plate 2			Plate 3		
Marker	Hetero	Small	Marker	Hetero	Small	Marker	Hetero	Small
CD1a	0.72	0.07	CD86	0.82	0.15	CD279	0.83	0.61
CD1b	0.84	0.26	CD87	0.61	2.79	CD282	0.71	0.25
CD2	0.42	0.14	CD88	0.42	0.28	CD305	0.72	0.22
CD3	0.24	0.17	CD89	0.56	0.3	CD309	1.14	0.33
CD4	0.34	0.14	CD90	99.66	99.19	CD314	0.92	0.21
CD4v4	0.46	0.42	CD91	55.04	51.93	CD321	1.43	0.41
CD5	0.1	0.12	CDw93	0.29	2.84	CDw327	0.7	0.17
CD6	0.32	0.14	CD94	0.7	0.48	CDw328	0.96	0.08
CD7	0.28	0.12	CD95	77.84	99.29	CDw329	1.56	0.2
CD8a	0.23	0.15	CD97	10.39	9.2	CD335	1.52	0.16
CD8b	0.23	0.16	CD98	99.79	99.74	CD336	1.27	0.13
CD9	74.66	81.48	CD99	98.64	99.83	CD337	2.13	0.45
CD10	1.01	10.81	CD99R	77.12	82.76	CD338	0.92	1.51
CD11a	0.93	0.18	CD100	0.99	0.61	CD340	31.23	98.68
CD11b	0.79	0.14	CD102	0.95	0.73	αβTCR	4.96	0.7
CD11c	0.34	0.12	CD103	0.44	0.25	β2microglobulin	88.67	99.65
CD13	97.2	97.09	CD105	99.88	99.89	BLTR-1	1.59	0.18
CD14	0.45	0.61	CD106	24.41	13.03	CLIP	1.05	0.26
CD15	0.09	0.12	CD107a	26.67	55.66	CMRF-44	0.53	0.3
CD15s	0.21	0.08	CD107b	15.55	5.45	CMRF-56	1.18	0.51
CD16	0.47	0.22	CD108	79.64	97.26	EGF receptor	45.39	96.48
CD18	0.44	0.18	CD109	95.79	99.58	fMLP receptor	1.91	0.74
CD19	0.26	0.18	CD112	14.1	29.51	γδTCR	2.03	0.34
CD20	0.52	0.24	CD114	0.97	0.39	Hem.Prog.Cell	5.13	1.59
CD21	1.03	0.2	CD116	0.94	0.48	HLA-A, B, C	99.62	99.79
CD22	0.67	0.19	CD117	0.94	0.26	HLA-A2	99.65	99.83
CD23	0.64	0.11	CD118	0.56	0.31	HLA-DQ	23.27	8.4
CD24	0.51	0.16	CD119	58.26	55.81	HLA-DR	0.71	0.35
CD25	0.42	0.16	CD120a	5.94	19.55	HLA-DR, DP, DQ	0.58	0.35
CD26	11.43	11.81	CD121a	1.8	1.86	Invariant NKT	1.13	0.2
CD26	11.43	11.81	CD121b	2.15	0.29	GD2[1]	54.94	9.98
CD27	0.59	0.22	CD122	6.25	0.49	MIC A/B	0.57	0.17
CD28	1.5	0.17	CD123	2.24	0.37	NKB1	1.22	0.2
CD29	97.54	98.34	CD124	3.84	0.57	SSEA-1	0.35	0.41
CD30	2.91	0.48	CD126	2.05	0.55	SSEA-4	52.44	67.01
CD31	0.5	0.29	CD127	0.47	0.43	TRA-1-60	0.61	0.76
CD32	0.5	0.59	CD128b	0.58	0.17	TRA-1-81	0.39	0.44
CD33	0.7	0.17	CD130	12.78	28.49	Vβ23	3.72	0.51
CD34	3.25	0.6	CD134	0.83	0.36	Vβ8	1.45	16.79
CD35	93.29	0.28	CD135	0.39	0.31	mIgM	0.19	0.25
CD36	0.66	0.24	CD137	0.43	0.23	mIgG1	0.3	0.14
CD37	1.2	0.16	CD137[2]	3.81	0.7	mIgG2a	0.18	0.24
CD38	0.99	0.23	CD138	87.76	92.86	mIgG2b	0.24	0.18
CD39	4.39	0.46	CD140a	76.81	97.18	mIgG3	0.36	0.49
CD40	1.23	0.42	CD140b	99.34	99.82	CD49f	26.58	75.93
CD41a	0.99	0.23	CD141	63.94	25.29	CD104	0.09	0.35

CD41b	0.15	0.04	CD142	2.45	3.56	CD120b	0.15	0.12
CD42a	7.59	0.80	CD144	1.34	0.53	CD132	0.12	0.35
CD42b	1.42	0.21	CD146	99.68	99.74	CD201	51.1	78.93
CD43	1.05	0.32	CD147	99.84	99.95	CD210	0.25	0.85
CD44	99.05	99.76	CD150	0.99	0.92	CD212	0.18	0.08
CD45	2.5	0.51	CD151	99.45	99.23	CD267	0.13	0.1
CD45RA	1.63	0.62	CD152	23.86	17.79	CD294	0.13	0.06
CD45RB	0.7	0.12	CD153	3.66	0.42	CD326	4.58	1.36
CD45RO	0.7	0.48	CD154	2.06	0.42	CLA[3]	0.28	0.18
CD46	97.77	99.58	CD158a	0.41	0.96	Integrin β7	0.1	0.06
CD47	97.71	99.88	CD158b	3.34	0.76	SSEA-3	0.78	0.4
CD48	6.98	0.74	CD161	1.81	0.45	rIgM	0.22	0.18
CD49a	90.67	47.74	CD162	3.77	1	rIgG1	0.16	0.08
CD49b	29.04	79.47	CD163	0.49	0.35	rIgG2a	0.18	0.05
CD49c	98.71	99.65	CD164	53.18	71.87	rRIgG2b	0.34	0.3
CD49d	48.73	72.21	CD165	98.17	99.74			
CD49e	99.26	99.84	CD166	99.72	99.91			
CD50	4.83	1.91	CD171	1.1	0.53			
CD51/61	89.52	96.28	CD172b	1.22	2.47			
CD53	1.67	0.28	CD177	1.87	0.58			
CD54	35.02	14.08	CD178	1.62	0.42			
CD55	86.08	95.08	CD180	6.44	0.5			
CD56	3.93	2.22	CD181	7.01	2.06			
CD57	1.18	0.47	CD183	6.09	0.81			
CD58	95.74	99.73	CD184	4.1	1.16			
CD59	99.76	99.91	CD193	2.41	1.65			
CD61	81.54	96.71	CD195	0.12	0.21			
CD62E	3.22	0.73	CD196	4	0.51			
CD62L	2.24	0.23	CD197	1.75	0.35			
CD62P	0.39	0.35	CD200	1.95	0.12			
CD63	71.8	97.95	CD205	0.14	1.1			
CD64	0.91	0.37	CD206	0.49	0.37			
CD66[4]	0.77	0.28	CD209	4.47	4.79			
CD66b	0.3	0.17	CD220	11.29	4.49			
CD66f	0.85	0.22	CD221	27.67	26.19			
CD69	0.62	0.2	CD226	2.94	0.38			
CD70	0.18	0.03	CD227	24.18	4.84			
CD71	41.71	50.17	CD229	0.3	0.39			
CD72	0.45	1.06	CD231	0.58	0.34			
CD73	99.63	99.49	CD235a	1.31	0.86			
CD74	0.84	0.82	CD243	3.76	1.69			
CD75	2.01	0.41	CD244	0.1	0.27			
CD77	4.54	6.62	CD255	0.29	0.3			
CD79b	8.77	0.68	CD268	1.57	0.75			
CD80	1.42	0.55	CD271	4.19	0.86			
CD81	99.24	99.49	CD273	91.5	99.77			
CD83	3.35	0.56	CD274	77.92	97.17			
CD84	0.73	0.34	CD275	3.9	1.74			
CD85	0.34	0.7	CD278	1.57	0.45			

[1] Disialoganglioside GD2, [2] CD137 ligand, [3] Cutaneous Lymph A, [4] CD166 (a, c, d, e)

Supplementary Table 5. The expression of EGFR and CD49f on small size cell during passaging as analyzed by flow cytometry.

	Expression (%)			
Passage	EGFR+CD49F+	EGFR+CD49f-	EGFR-CD49f+	EGFR-CD49f-
3	70	9	8	13
5	52	15	7	25
7	40	11	4	45
